# Laser-Induced Modification of Hydrogenated Detonation Nanodiamonds in Ethanol

**DOI:** 10.3390/nano11092251

**Published:** 2021-08-31

**Authors:** Irena Bydzovska, Ekaterina Shagieva, Ivan Gordeev, Oleksandr Romanyuk, Zuzana Nemeckova, Jiri Henych, Lukas Ondic, Alexander Kromka, Stepan Stehlik

**Affiliations:** 1 Institute of Physics of the Czech Academy of Sciences, Cukrovarnická 10, 16200 Prague, Czech Republic; bydzovska@fzu.cz (I.B.); shagieva@fzu.cz (E.S.); gordeev@fzu.cz (I.G.); romanyuk@fzu.cz (O.R.); ondic@fzu.cz (L.O.); kromka@fzu.cz (A.K.); 2 Faculty of Nuclear Sciences and Physical Engineering, Czech Technical University in Prague, Břehová 7, 11519 Prague, Czech Republic; 3 Institute of Inorganic Chemistry of the Czech Academy of Sciences, 25068 Husinec-Řež, Czech Republic; nemeckova@iic.cas.cz (Z.N.); henych@iic.cas.cz (J.H.); 4 Faculty of Environment, J.E. Purkyně University in Ústí nad Labem, Pasteurova 3632/15, 40096 Ústí nad Labem, Czech Republic

**Keywords:** nanodiamond, onion-like carbon, carbon nano-onion, laser, surface chemistry, structure, zeta potential, Raman spectroscopy

## Abstract

Apart from the frequently used high-temperature annealing of detonation nanodiamonds (DNDs) in an inert environment, laser irradiation of DNDs in a liquid can be effectively used for onion-like carbon (OLC) formation. Here, we used fully de-aggregated hydrogenated DNDs (H-DNDs) dispersed in ethanol, which were irradiated for up to 60 min using a 532 nm NdYAG laser with an energy of 150 mJ in a pulse (5 J/cm^2^) at a pulse duration of 10 ns and a repetition rate of 10 Hz. We investigated the DND surface chemistry, zeta potential, and structure as a function of laser irradiation time. Infrared spectroscopy revealed a monotonical decrease in the C–H_x_ band intensities and an increase of the C–O and C=O features. Transmission electron microscopy (TEM) revealed the formation of OLC, as well as a gradual loss of nanoparticle character, with increasing irradiation time. Surprisingly, for samples irradiated up to 40 min, the typical and unchanged DND Raman spectrum was recovered after their annealing in air at 450 °C for 300 min. This finding indicates the inhomogeneous sp^3^ to sp^2^ carbon transformation during laser irradiation, as well as the insensitivity of DND Raman spectra to surface chemistry, size, and transient structural changes.

## 1. Introduction

Onion-like carbon (OLC) is a spherical, carbon-based nanomaterial that, in ideal cases, consists of concentric layers formed by sp^2^-hybridized carbon atoms. From this point of view, it is a spherical analog of multi-walled carbon nanotubes. Recently, OLCs have attracted great attention for energy harvesting [1] and storage [2], as well as catalysis [3] or bioimaging [4]. In these applications the use of OLCs is contingent on their good electrical conductivity, light absorption/emission properties, and high surface area. Various reports in the literature have shown that the tailoring of such properties is indeed feasible. 

There are numerous methods of synthesis of OLCs, such as the arc discharge process, flame synthesis, pyrolysis, and nanodiamond (ND) transformation at high temperature (>1000 °C) [5]. Detonation nanodiamonds (DNDs) with sub-10 nm size and a relatively narrow size distribution, peaking around ~4–5 nm, represent a suitable precursor for high-yield and industrial-scale OLC synthesis [6]. The temperature-induced transformation of NDs to OLCs under vacuum or inert gas (e.g. Ar) proceeds in several stages. First, the desorption of surface functional groups occurs at temperatures of 500–700 °C [7,8]. The process continues by formation of surface-graphitized NDs above 700 °C [9]. Next, when the temperature rises above 1000 °C, the external graphene patches start to merge and the defect density decreases [10]. Highly crystalline and low-defect OLCs are typically obtained at annealing temperatures higher than 1500 °C [11]. At this temperature, the complete DND-to-OLC transformation lasts tens of minutes [12]. Such created OLCs are purely graphitic, with minimal or even no occurrence of surface functional groups consisting of foreign atoms such as oxygen. This makes the OLCs hydrophobic and limits their use in applications where water solubility is needed, e.g., in biological applications. High temperature annealing could also facilitate sintering of the primary ND particles and a decrease of specific surface area. 

Laser irradiation has found extended applications in the controlled manipulation of materials for electrochemical energy storage and conversion [13]. Laser processing can be used for modification of broad band gap materials such as diamond in bulk or thin film forms [14,15], as well as in the nanoparticle form of NDs. It has been shown that laser irradiation (ns pulses) of DND powder dispersed in a liquid can be used to modify the DND surface in terms of Csp^2^ content or surface functional groups [16]. It is also a convenient method of preparing alcohol-soluble OLCs with remarkable electrocatalytic activity [3,17]. Compared to the vacuum or inert gas annealing methods (i.e., transformation at high temperature), the laser irradiation is highly non-equilibrium process due to the ns duration of the pulses. This, in general, lowers the probability of the formation of well-ordered crystalline structures. Ambient conditions and the presence of a surrounding medium, i.e., a solvent containing or constituting dissolved oxygen, provide the OLCs with surface oxygen-containing functional groups via a photo-induced reaction between the NDs and the solvent. If the laser irradiation is not stopped at the right time, the formed OLC are gradually photo-oxidized until they completely disappear [3]. Surprisingly, instead of photooxidation [3], Xiao et al. reported a reversible ND-OLC phase transformation using a very similar experimental arrangement, though with a higher laser pulse energy [18]. Such results are also potentially very interesting from an ND synthesis point of view. One can imagine that great control over the ND properties (doping, size, and structure) could be achieved by using well-defined carbon precursors, such as carbon dots [19]. 

In this work we employed de-aggregated, hydrogenated ~5 nm DNDs dispersed in ethanol to investigate the surface chemistry evolution and structural changes during the laser-induced graphitization of individual DND particles. Hydrogenated DNDs have several advantages. In contrast to oxidized DND, the surface chemistry of hydrogenated DNDs is simple and relatively well understood [20]. It is oxygen-free and thus provides a good basis for the investigation of the photooxidation reaction. The hydrogenated DNDs are also well soluble in ethanol, which has been shown to be a good solvent for OLC synthesis, in contrast to water [3]. Finally, the motivation for this work was to reproduce the reversible OLC to ND-OLC phase transformation reported by Xiao et al. [18] and possibly to clarify the inconsistent results reported so far [3,18]. However, as presented in this paper, the reverse transformation of OLC-ND seems not to be valid.

## 2. Materials and Methods

Fully de-aggregated DNDs in powder form (FDP; fumed diamond powder, NanoCarbon Research Institute, Ueda, Japan) were hydrogenated by annealing in hydrogen gas at 700 °C for 6 h at atmospheric pressure with hydrogen providing fully hydrogenated H-DNDs [21]. Then, 20 mg of H-DNDs were dispersed in 10 mL of ethanol with 1 h sonication using an ultrasonic processor (Hielscher UP 200s; Teltow, Germany) working at 100% amplitude and a 50% on/off period. After the sonication, the H-DND solution in ethanol was diluted to the final concentrations and used for the laser irradiation.

A Q-switched Nd YAG laser (Ekspla, Vilnius, Lithuania) with a wavelength of 532 nm, pulse width of 10 ns, repeating frequency of 10 Hz, and pulse energy of 150 mJ was used for irradiation of 6 mL of H-DNDs (1.2 mg/mL) dispersed in ethanol. A laser beam with a diameter of 2 mm was sent through the central part of a clear glass bottle filled with the H-DND solution, which yielded the laser fluence of ~5 J/cm^2^. The solution was continuously stirred with a magnetic stirrer during the irradiation process. The irradiation lasted for 1, 2, 3, 4, 5, 10, 20, 40, and 60 min, providing nine samples. The processed samples were then dried by evaporation and re-dispersed in DI water by means of the same sonication treatment as mentioned above. To reproduce the reversible nanodiamond–carbon onion phase transformations we also prepared a second sample set according to Xiao et al. [18]. Therefore, 3 mg of raw DND powder (New Metals and Chemicals; Tokyo, Japan) was dispersed in 10 mL of ethanol by 1 h sonication, as above. Such prepared samples were irradiated with a laser beam with the same parameters as above, except for the diameter, which was reduced down to 1 mm to reach the same laser fluence (20 J/cm^2^) as used in the work [18]. 

The zeta potential of pristine H-DNDs and 5 and 10 min water-based samples was measured in disposable cuvettes by electrophoretic velocity measurement using a dynamic light scattering system (Zetasizer nano ZS; Malvern Panalytical; Malvern, UK). Each sample was measured until a million counts were reached, to achieve a good signal-to-noise ratio. 

Fourier transform infrared (FTIR) spectra were measured using an N_2_-purged spectrometer (Nicolet iS50; Thermo Fisher Scientific, Waltham, MA, USA) equipped with a KBr beam splitter and N_2_-cooled MTC-HighD* detector. All spectra were measured by a specular apertured grazing angle reflectance method with an 80° incident light. Each spectrum represents an average of 128 scans per spectrum collected with a resolution of 4 cm^−1^. The pristine H-DNDs and 5 and 10 min water-based samples were drop-casted (50 μL) on gold-coated Si substrates, and the bulk water was evaporated at 100 °C for 1 min. A bare gold-coated Si substrate was measured as a background prior to each sample measurement.

Samples for X-ray photoelectron spectroscopy (XPS) analysis were prepared in the same way as for the FTIR measurements and after evaporation of the water were introduced into an AXIS-Supra photoelectron spectrometer (Kratos Analytical Ltd., Stretford, UK). The thickness of the samples was more than 10 nm, but in some cases the Au 4f spectra from the Au substrates were also detected. Survey spectra and high-resolution core-level spectra were measured using monochromatized Al Kα radiation (incidence photon energy was 1486.6 eV; exposed X-ray spot size was 0.7 × 0.3 mm^2^). Photoelectrons were collected in a constant analyzer energy mode with a pass energy of 10 eV, resulting in an overall energy resolution of 0.45 eV (measured on the Ag 3d^5/2^ line width). The X-ray incidence angle was 54.4°, and the photoelectron emission angle was 0° with respect to the surface normal. All spectra were calibrated to the binding energy of the sp^3^ phase in the C 1s peak at 285 eV. Atomic concentrations were determined from the corresponding photoelectron peak areas after standard Shirley inelastic background subtraction and considering the relative sensitivity factors of elements (implemented in ESCApe software). High-resolution spectra were fitted by the KolXPD program using Gaussian functions. 

Scanning electron microscopy (SEM) images were obtained at 15 kV and a magnification of 300,000× in the regime of secondary electrons (MAIA 3, Tescan, Brno, Czech Republic). The samples were prepared by the drop-casting of water-based samples on Si substrates and drying at 100 °C for 1 min.

Transmission electron microscopy (TEM) images and electron diffraction patterns were acquired using an FEI Talos F200X (Thermo Fisher Scientific, Waltham, MA, USA) microscope. The samples were prepared by drop-casting of water-based samples on a conventional holey-carbon film-coated copper grid.

Raman spectra were obtained with a InVia system (Renishaw, Wotton-under-Edge, UK) equipped with a 325 nm He-Cd excitation laser (Kimmon Koha, Tokyo, Japan). The samples were prepared by drop-casting on Si substrates. The laser power did not exceed 1 mW. The samples were purified from the laser-created sp^2^-C by annealing in air on the Si substrates. The annealing at 450 °C lasted 30, 90, and 300 min.

## 3. Results and Discussion

### 3.1. Visual Appearance Evolution

**Figure 1:**
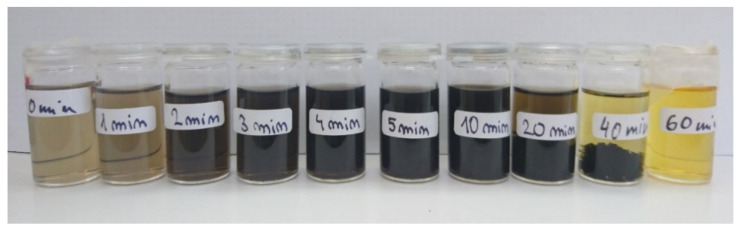
Optical photograph of pristine and laser treated H-DNDs in ethanol up to 60 min.

Figure 1 shows the distinct visual change of the samples as a function of the laser irradiation time. The brownish color and transparency of the pristine, non-irradiated H-DND colloid indicates well-dispersed single-digit H-DNDs [22]. With the increase of the laser irradiation time, the brown color gradually changes to black (5, 10 min) and finally to transparent yellow (60 min). The color evolution perfectly correlates with the previous studies [3,18]. The samples up to 10 min of irradiation are homogeneous but sedimentation of a black precipitate occurs for the 20 min and 40 min samples. The 60 min yellow sample is transparent, with only residual black solids. Analyses of the surface chemistry and structure of the pristine and irradiated samples are provided below.

### 3.2. Infrared Spectroscopy

**Figure 2:**
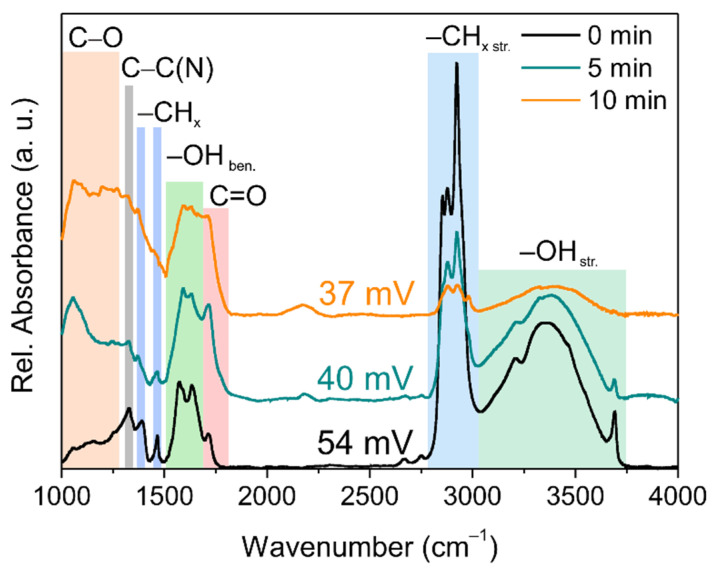
FTIR spectra of the pristine H-DND (0 min) and H-DND irradiated for 5 and 10 min.

Fourier-transform infrared spectroscopy (FTIR) was used to characterize the surface chemistry evolution with an increasing time of laser irradiation. Figure 2 shows the FTIR spectra of the pristine H-DNDs and laser irradiated (5 and 10 min), together with the ZP values noted above a particular spectrum. The FTIR spectrum of the pristine H-DNDs shows typical features of hydrogenated DNDs, i.e., mainly strong C–H_x_ stretching (2800–3000 cm^−1^) and C–H_x_ bending (1380 and 1465 cm^−1^) features. The unusual hydration of the H-DNDs [23,24] gives rise to a multicomponent OH bond structure in the bending (1500–1700 cm^−1^) as well as stretching (3000–3750 cm^−1^) region, including a free OH vibration peak at 3695 cm^−1^, which is characteristic for hydrogenated DNDs [23,24]. The feature at around 1720 cm^−1^ appears due to residual C=O bonds. Finally, the sharp peak at around 1330 cm^−1^ comes from the Raman-like C–C vibrations and is typically inactive in IR. This band corresponds to the defect-induced one-phonon mode enabled by embedded tetrahedral substitutional nitrogen originating from the nitrogen-rich explosives commonly used for detonation synthesis of NDs [25]. The appearance of this volume-related band in the FTIR spectrum can be understood as a “fingerprint” of the DND structure [20]. The preservation of the 1330 cm^−1^ peak up to 10 min of laser irradiation indicates that the DND structure is still present, most probably in the interior of already graphitized particles. However, other spectral features exhibit a significant evolution with increasing irradiation time. The most obvious is the decrease of C–H_x_ bond intensity (both stretching and bending) with increasing time of laser irradiation. Second, the C=O feature increases with the intensity, and a new C–O feature at around 1050 cm^−1^ appears. This indicates photoinduced oxidation of the surface C–H_x_ bonds, which is in agreement with the previous study [3].

The photooxidation is accompanied by a decrease of the zeta potential. Zeta potential is an indicator of colloidal stability, and its decrease signalizes a change in surface chemistry. We were able to obtain ZP values only from the colloidally homogeneous samples up to 10 min of irradiation. The initially highly positive ZP of the pristine H-DNDs steadily decreased with irradiation time to a value close to a colloidal stability limit of 30 mV. Highly positive ZP of H-NDs is a well-known phenomenon but still not completely understood. Specifically, in H-DNDs this phenomenon is assigned to the basicity and protonation of the H-DND sp^2^ carbon surface [26]. It is not yet clear, however, to what extent the presence of diamond core is important for a positive ZP, since hydrogenated or pure sp^2^ carbon nanostructures are hydrophobic and do not form stable colloids in water [27]. Thus, that the ZP decreases with the irradiation time could, in principle, reflect both the structural and surface chemistry changes of the H-DNDs.

### 3.3. X-ray Photoelectron Spectroscopy

The surface chemistry/structural (changes in hybridization of carbon atoms) changes induced by the laser irradiation were further investigated by XPS. The chemical compositions of the pristine H-DNDs and the H-DNDs irradiated for 5, 10, 20, and 40 min are summarized in Table 1. The pristine H-DNDs sample contains only 2.0 at. % oxygen, originating from residual surface-adsorbed water and/or residual surface oxygen-containing groups. The oxygen concentration significantly increased with laser irradiation time up to approx. 18.2 at. % for the 40 min sample. This correlates well with the FTIR and ZP analysis and confirms gradual photo-oxidation of the H-DNDs with increasing irradiation time. Apart from dominant carbon, the XPS analysis also revealed the presence of nitrogen (1.6 % at. %) as a relic of the detonation synthesis, as well as trace amounts of Zr, Na, and Cl most probably originating from the ZrO_2_ microbead milling and purification of the used FDP material. The concentration of these contaminants was below 0.5 at. % (less than the error bars of the measurements). Therefore, contaminants are not included in Table 1.

**Figure 3:**
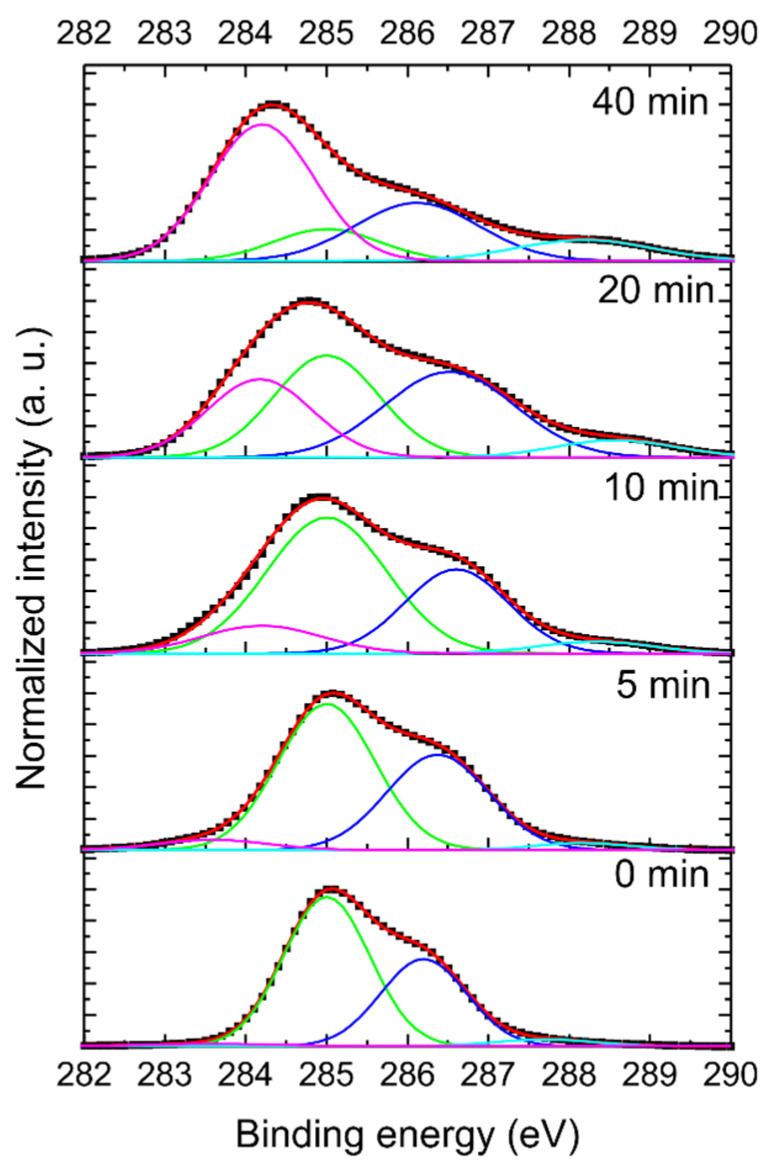
C 1s spectra of the pristine H-DND sample (0 min) and laser-irradiated H-DNDs samples. The irradiation time (5, 10, 20, and 40 min) is indicated. Experimental data points are shown as black squares, overall spectra fits as red lines, and the particular bond components as green (sp^3^), magenta (sp^2^), blue (C–N, C_def_), and cyan (C=O).

Figure 3 depicts the deconvoluted C 1s spectrum of the pristine H-DND, and the H-DNDs laser-irradiated for 5, 10, 20, and 40 min. The C 1s spectrum of the pristine sample consists of two prominent components, peaking at 285.0 eV (green) and at 286.2 eV (blue). While the green originates from the sp^3^ hybridized C, the blue was assigned to a defective, C_def_, carbon sp^3^ phase and/or C–N bonds [8] and has been shown to be characteristic for DND [28]. Contributions from sp^2^ hybridized C (magenta) and C=O (cyan) were negligible in the pristine sample. With the increase of the laser irradiation time, the intensity of the sp^2^ component of the C 1s increases, resulting in an increase of the sp^2^/sp^3^ ratio, as summarized in Table 1. A noticeable increase is also observed in the C_def_/sp^3^ component ratio of the C 1s peak. This trend clearly documents a gradual change of the DND structure: the sp^3^ dominant carbon phase is transformed to the sp^2^ carbon phase by the laser irradiation, whereas the C–N/ C_def_ bonds are more resistive to the laser-induced structural changes. It is also possible, however, that Csp^3^-N to Csp^2^-N re-bonding occurred, but we were not able to resolve such changes due to the very low N concentrations and component intensities. Nevertheless, even after 40 min of laser irradiation, the structural transformation was not completed, and some sp^3^ carbon phase remained detectable in the sample. 

### 3.4. Electron Microscopy

**Figure 4:**
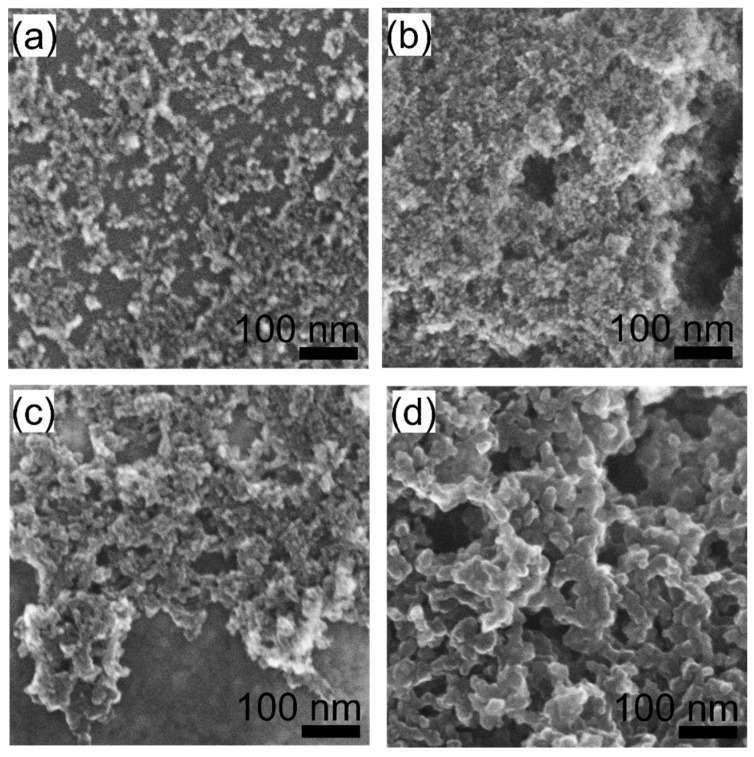
SEM images of pristine (**a**) and laser irradiated H-DNDs for 10 min (**b**), 20 min (**c**), and 40 min (**d**).

To investigate the nanoscale morphology and its evolution with irradiation time, we performed electron microscopy analysis. SEM images of the 0, 5, 10, 20, and 40 min samples drop-casted on the Si substrate are shown in Figure 4. The SEM image of the pristine sample reveals a typical DND nanoparticle-like morphology; although the DND particles form aggregates upon drying. With increasing irradiation time, the nanoparticle character gradually disappears, and a coarsening of the structure becomes apparent and larger units appear. The 40 min sample is no longer formed by single digit particles (<10 nm), but instead a fiber-like nanostructure is observed.

**Figure 5:**
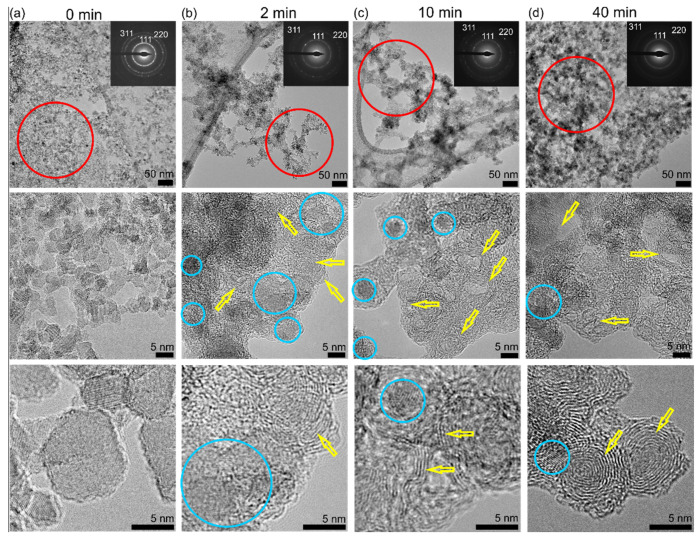
TEM images and electron diffraction patterns of the pristine (0 min) H-DNDs (**a**), and samples irradiated for 2 min (**b**), 10 min (**c**), and 40 min (**d**). The yellow arrows indicate the laser irradiation-created OLC structure, the blue circles denote the diamond crystalline structure.

More detailed information about the structure and phase composition was obtained by TEM. Figure 5 shows TEM images of the pristine, 2 min, 10 min, and 40 min samples at lower magnification (upper row) and at higher magnification (middle row), up to atomic resolution (bottom row). 

TEM analysis of the pristine H-DND sample reveals a typical, well-dispersed 2–10 nm DND particles. In agreement with recent reports, the larger particles (>5 nm) were often faceted, while the smaller particles showed a lower degree of faceting [20,29]. The higher magnification image shows that the H-DND surface is rather clean, without any sign of an ordered graphitic structure. The electron diffraction patterns confirm the diamond crystalline structure with 2.051 Å, 1.252 Å, and 1.069 Å lattice spacing for the 111, 220, and 311 diffractions, respectively. Already after 2 min of laser irradiation, particles with an ordered graphitic shell (denoted by a yellow arrow) appear, together with an amorphous material. The diamond crystalline structure (blue circles) is still present, as was also confirmed by the electron diffraction pattern. The sample irradiated for 10 min exhibited a higher number of particles with an ordered carbon shell than the 2 min sample. After 40 min of irradiation, well-ordered OLCs could be found. However, in all the irradiated samples, even after 40 min of irradiation, a diamond crystalline structure was still present (blue circles), as also confirmed by the electron diffraction pattern and the XPS analysis above. The low magnification TEM images corroborate the SEM images, revealing a coarsening, interconnection, and overall size increase of the objects in the irradiated samples. Formation of an interconnected structure could have been promoted by the specific experimental conditions. FTIR and XPS revealed that laser irradiation leads to gradual photooxidation of the surface C–H_x_ groups, which is accompanied by a decrease of the zeta potential. Since the laser hits only a small discrete volume in one pulse, the irradiation is inhomogeneous, and some H-DNDs may become more oxidized than others. This could promote the electrostatic attraction between more and less oxidized H-DNDs, resulting in the formation of aggregates. With ongoing laser irradiation, the interparticle bonds in aggregates become stronger (“sintering”), and finally the DND particles merge with one another and are transformed a to fiber-like C-sp^2^ nanostructure. Thus, the surface chemistry of the used DND could potentially be used to control the morphology of the obtained graphitic product.

### 3.5. Raman Spectroscopy

**Figure 6:**
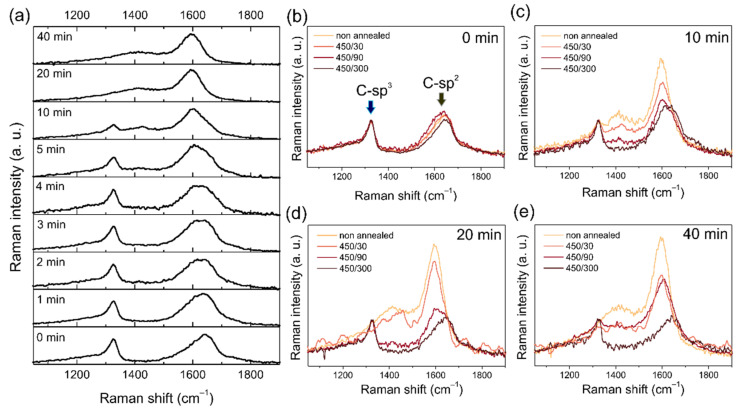
UV Raman spectra of laser irradiated DNDs up to 40 min (**a**). UV Raman spectra of laser treated DNDs for 0 min (**b**), 10 min (**c**), 20 min (**d**), and 40 min (**e**), without annealing, after annealing in air at 450 °C for 30 min, 90 min, and 300 min. Due to the high noise level in the 450/30 spectra of 20 min (**d**) and 40 min (**e**), smoothing with the adjacent averaging method was applied.

Raman spectroscopy is a powerful technique for investigating the allotropic composition of carbon materials. In the Raman spectroscopy investigation, we focused on two aspects. First, we used Raman spectroscopy to confirm the laser induced transformation of the H-DND into graphitic structures such as OLCs [3], and possibly back into NDs [18]. Second, we used Raman spectroscopy to characterize the samples after the air annealing treatments aimed at selective purification of the sp^2^ carbon [30] created by the laser irradiation. The Raman spectra of all the studied samples as a function of irradiation time are shown in the Figure 6a. The Raman spectrum of the pristine H-DNDs shows typical features of detonation nanodiamond, i.e., a broadened and shifted peak of diamond at around 1327 cm^−1^ accompanied by a low frequency shoulder, as well as a broad feature with a maximum around 1640 cm^−1^, often denoted as a G-band [31]. It is immediately apparent that with increasing the irradiation time up to 40 min the maximum of the G-band shifts down to ~1600–1610 cm^−1^. The signal at 1640 cm^−1^ in purified DND probably comes from sp^2^ fragments or chains [31]. With increasing irradiation time (and gradual graphitization) larger sp^2^ domains are formed. This is accompanied by an increase of the new band at around 1400 cm^−1^, which is assigned to D-band, i.e., the defect-related signal from graphitic carbon. At the same time, the diamond peak gradually disappears and it is no longer detectable from 20 min onwards. Such an evolution correlates with the optical photograph, XPS data, and SEM/TEM images, i.e., a gradual, laser induced C-sp^3^ to C-sp^2^ structural transformation occurs. In the 60 min sample, no signal of nanodiamonds was detected, which is contradictory to the previous report of reversible nanodiamond-carbon onion phase transformations [18]. Moreover, the deposit formed by drop-casting of the 60 min sample was very volatile, which, together with the FTIR analysis (Figure S1), supports the assumption that polyyne (C_n_H_2_) molecules could be the final stage of the photooxidation process [3]. 

The Raman spectra of the pristine H-DND and 10, 20, and 40 min samples after air annealing at 450 °C for 30, 90, and 300 min are shown in Figure 6b–e. The purification effect achieved by annealing in air at 450 °C on the pristine H-DND sample is noticeable but not very pronounced. There is only a minor decay of the G-band intensity at the shorter wavenumber side (around 1600 cm^−1^) with increasing annealing time. Other spectral features of the annealed samples remained identical to the pristine H-DND. In contrast, the purification effect of air annealing at 450 °C was clearly evident in the laser treated samples. The C-sp^2^ material created by the laser irradiation, manifested by the D-band and G-band features, was selectively burned during air annealing [30] and thus, the intensity of the related spectral features was recognizably reduced. 

Regardless of the laser irradiation time, the typical DND spectrum (and HRTEM image, see Figure S2) was always retrieved after the longest air annealing, for 300 min at 450 °C. This clearly confirms that even after 40 min of laser treatment, not all the DNDs were transformed to an OLC-like structure. At the same time, this endorses that the G_DND_-band is an unavoidable part of the DND Raman spectrum, regardless of size, purity, and surface chemistry [28,31,32,33]. This clearly questions previous assignments of the G_DND_-band as a DND surface-related feature and supports a recently proposed scenario, whereby at least part of the G-band signal is related to the DND volume [28]. As documented here using TEM, and also in previous studies, the DND graphitization starts from the surface and continues to the particle volume [12]. Therefore, the air-annealed DND are unavoidably modified by (i) graphitization of its surface, i.e., formation of a C-sp^2^ shell, whose thickness depends on the laser irradiation time; followed by (ii) subsequent and preferential removal of C-sp^2^ by the air annealing purification. Taking into account the relatively narrow DND size distribution, it can be supposed that, in general, these modifications reduce the DND particle mean size, which should be reflected in the Raman spectrum [34,35], namely in the diamond peak line shape parameters. This was not observed here, which agrees with the previous reports highlighting the size-insensitivity of the DND Raman spectra [28]. 

The evolution of the Raman spectra confirms, in general, the gradual C-sp^3^ to C-sp^2^ transformation. As intermediate states, the hybrid C-sp^3/2^ nanostructures [8] can be obtained, as also confirmed by XPS and TEM. Such structures are potentially interesting for carbocatalysis, since the C-sp^3/2^ nanostructures can benefit from a unique electronic structure [36]. This involves the graphitic shell providing the properties of C-sp^2^ with a conjugated π-electron system. It is expected that the inner diamond core is capable of tailoring the surface electron states of the C-sp^2^ shell by directly injecting electrons via the covalent bonds at the graphene/diamond interfaces [37].

The underlying mechanisms of the DND graphitization and photooxidation by 532 nm laser irradiation with ns pulses in a liquid are not completely described. Although pure bulk diamond has a broad band gap energy of 5.45 eV and does not absorb the 532 nm green light, DND are far from being pure and perfect diamond nanocrystals. Instead, they absorb visible light, which leads to their heating during 532 nm laser irradiation. Up to this point, the graphitization process is analogous to the vacuum or inert gas annealing of DNDs [6,8,12]. The OLC structure formation starts from the DND surface inward. Before the graphitization starts, the surface functional groups desorb, which activates the surface reactivity, and the created surface dangling bonds connect with one another, forming into graphene-like shells in ideal cases, i.e., with no terminal surface chemical groups. 

Moreover, the reactive surface also promotes local sintering and connections between individual particles [38]. From this point of view, the graphitization of individual DNDs in a colloid and preservation of their nanoparticle nature would be beneficial. Our results show that under the used conditions, such an aim is indeed partially achievable, when colloidally stable, partially graphitized DNDs are obtained up to 10 min of irradiation. However, complete graphitization was not achieved, even after 40 min of irradiation, i.e., at the point where the initial nanoparticle character had disappeared (see Figure 4 and Figure 5).

In the case where DNDs are not in an inert gas or vacuum, but in a liquid in the form of a colloid, both solvent and DNDs are inevitably involved in the processes. For instance, it is the ND surface reactivity which facilitates the hydrogenation of their surface by annealing in hydrogen gas at a relatively low temperature (500 °C and higher) [7]. It was suggested in the previous study that solvent oxidation ability is an important parameter that controls the OLC formation rate [3]. However, other solvent properties such as specific heat capacity cannot be excluded. Alternatively, the much higher specific heat capacity of the water (4180 J kg^−1^ K^−1^) compared to alcohols (methanol; 2500 J kg^−1^ K^−1^) could contribute to the lower transformation rate observed for water. In any case, since the DNDs are magnetically stirred in a liquid and new surface bonds are formed during every laser pulse, a repetition of this process combined with DND movement eventually leads to complete photo-oxidation and disappearance of the OLC-like structure. It needs to be pointed out that the graphitization is probably a self-accelerating process, i.e., the more graphitic the structure, the more light is absorbed, and the faster the process proceeds. The ns nature of the laser pulses also localizes and determines the thermodynamic conditions of the C-sp^3^ to C-sp^2^ transformation, which are, however, very far from the thermodynamic equilibrium. This probably results in a less ordered graphitic structure than can be achieved by the thermal process (i.e., bulk-like process).

The temperature locally achieved in the samples depends on the physical properties of the DNDs and on the laser fluence; the energy per pulse per cm^2^. However, the exact dependence cannot be trivially assumed. In our case, the energy fluence of the laser beam was approximately 5 J/cm^2^, but due to the absorption and scattering of the laser beam while passing through the sample, the fluence decreases as a function of the depth in liquid. This effect is particularly pronounced for the stages when the sample is black-colored. This means that only a negligible amount of the sample is subject to this specific fluence, and the rest, which is in the path of the laser beam, absorbs less energy. The importance of the exact value of the fluence for the process of photooxidation described above is thus questionable. In order to determine the dependence of the process on the irradiation fluence, we increased the laser fluence to 20 J/cm^2^. Employing a similar type of samples as in Ref. [18], the transformation from ND to OLC was obtained, but in a shorter time period, as presented in detail in Supp. Information (Figure S3). The transformation from OLC back to ND, described in Ref. [18], was not observed in this work. Therefore, we point out the fact that when a certain level of fluence is reached, then the process of OLC formation and photooxidation will take a similar course. The value of fluence above this level only defines the speed of the transformation process from the NDs to the final yellowish solution. 

## 4. Conclusions

We investigated the surface chemical and structural modifications of colloidal DNDs induced by laser irradiation. For the laser irradiation (532 nm, 10 ns, 5 J/cm^2^ or 20 J/cm^2^), single-digit and fully hydrogenated DNDs dispersed in ethanol were used. With increasing laser irradiation time, we observed the gradual oxidation of the H-DND’s surface C–H_x_ bonds, a decrease of the initially highly positive zeta potential, and a loss of colloidal stability after 10 min of laser irradiation. At the same time, we evidenced the formation of an OLC-like structure, whose initially discrete nanoparticle-like character (up to 10 min) progressively evolved to an interconnected, fiber-like C-sp^2^ nanostructure (20–40 min), which finally disappeared after 60 min of laser irradiation by the photo-oxidation mechanism. No reversible nanodiamond–carbon onion phase transformation was observed, even at the higher laser fluence (20 J/cm^2^), which contradicted previous results. Instead we showed that the characteristic DND structure can persist in the samples with up to 40 min of laser irradiation and can be retrieved by selective purification from the sp^2^ carbon by annealing in air at 450 °C. In any case, the typical DND Raman spectrum did not exhibit any significant evolution or change compared to the pristine DND, which confirms the DND structure insensitivity to surface chemistry, size, and transient structural changes. Our results demonstrate the potential and limits of the laser irradiation technique as a tool for the formation of water-soluble OLCs, as well as bringing deeper understanding of DNDs, OLCs, and the relations between these two carbon-based nanomaterials.

## Figures and Tables

**Table 1:** Atomic composition, Csp^2^/Csp^3^, and C_def_/Csp^3^ ratios of the samples as a function of laser irradiation time.

	Atomic Concentration (±0.5 at. %)				
Sample	C	O	N	sp^2^/sp^3^	C_def_/sp^3^
0 min	96.4	2.0	1.6	0.03	0.59
5 min	94.6	3.8	1.6	0.14	0.77
10 min	89.5	9.2	1.3	0.20	0.53
20 min	84.2	15.0	0.8	0.77	1.05
40 min	81.4	18.2	0.4	4.3	2.15

## Data Availability

Data are available on reasonable request from the corresponding author.

## Supplementary Materials

The following are available online at https://www.mdpi.com/article/10.3390/nano11092251/s1, Figure S1: FTIR spectra of pristine H-DND (0 min; black) dispersed in ethanol and the final yellow solution obtained after 60 min, Figure S2 HRTEM image of the residual DNDs for the 40 min sample after air annealing purification, Figure S3: Photograph and Raman spectra of the pristine and laser-irradiated samples with a fluence of 20 J/cm^2^.

## References

[1] Mohapatra D., Nemala S.S., Sayed M.S., Shim J.-J., Mallick S., Bhargava P., Parida S. (2020). Carbon Nano-Onion-Powered Optically Transparent and Economical Dye-Sensitized Solar Cells. Nanoscale.

[2] Pech D., Brunet M., Durou H., Huang P., Mochalin V., Gogotsi Y., Taberna P.-L., Simon P. (2010). Ultrahigh-Power Micrometre-Sized Supercapacitors Based on Onion-like Carbon. Nat. Nanotechnol..

[3] Jang D.M., Im H.S., Back S.H., Park K., Lim Y.R., Jung C.S., Park J., Lee M. (2014). Laser-Induced Graphitization of Colloidal Nanodiamonds for Excellent Oxygen Reduction Reaction. Phys. Chem. Chem. Phys..

[4] Sonkar S.K., Ghosh M., Roy M., Begum A., Sarkar S. (2012). Carbon Nano-Onions as Nontoxic and High-Fluorescence Bioimaging Agent in Food Chain—An In Vivo Study from Unicellular *E. Coli* to Multicellular *C. Elegans*. Mater. Express.

[5] Zeiger M., Jäckel N., Mochalin V.N., Presser V. (2016). Review: Carbon Onions for Electrochemical Energy Storage. J. Mater. Chem. A.

[6] Aleksenskii A.E., Baidakova M.V., Vul A.Y., Davydov V.Y., Pevtsova Y.A. (1997). Diamond-Graphite Phase Transition in Ultradisperse-Diamond Clusters. Phys. Solid State.

[7] Ahmed A.-I., Mandal S., Gines L., Williams O.A., Cheng C.-L. (2016). Low Temperature Catalytic Reactivity of Nanodiamond in Molecular Hydrogen. Carbon.

[8] Petit T., Arnault J.-C., Girard H.A., Sennour M., Bergonzo P. (2011). Early Stages of Surface Graphitization on Nanodiamond Probed by X-ray Photoelectron Spectroscopy. Phys. Rev. B.

[9] Petit T., Arnault J.-C., Girard H.A., Sennour M., Kang T.-Y., Cheng C.-L., Bergonzo P. (2012). Oxygen Hole Doping of Nanodiamond. Nanoscale.

[10] Schüpfer D.B., Badaczewski F., Peilstöcker J., Guerra-Castro J.M., Shim H., Firoozabadi S., Beyer A., Volz K., Presser V., Heiliger C. (2021). Monitoring the Thermally Induced Transition from Sp^3^-Hybridized into Sp^2^-Hybridized Carbons. Carbon.

[11] Dhand V., Yadav M., Kim S.H., Rhee K.Y. (2021). A Comprehensive Review on the Prospects of Multi-Functional Carbon Nano Onions as an Effective, High- Performance Energy Storage Material. Carbon.

[12] Mykhaylyk O.O., Solonin Y.M., Batchelder D.N., Brydson R. (2005). Transformation of Nanodiamond into Carbon Onions: A Comparative Study by High-Resolution Transmission Electron Microscopy, Electron Energy-Loss Spectroscopy, X-ray Diffraction, Small-Angle X-ray Scattering, and Ultraviolet Raman Spectroscopy. J. Appl. Phys..

[13] Hu H., Li Q., Li L., Teng X., Feng Z., Zhang Y., Wu M., Qiu J. (2020). Laser Irradiation of Electrode Materials for Energy Storage and Conversion. Matter.

[14] De Feudis M., Caricato A.P., Taurino A., Ossi P.M., Castiglioni C., Brambilla L., Maruccio G., Monteduro A.G., Broitman E., Chiodini G. (2017). Diamond Graphitization by Laser-Writing for All-Carbon Detector Applications. Diam. Relat. Mater..

[15] Cadot G.B.J., Thomas K., Best J.P., Taylor A.A., Michler J., Axinte D.A., Billingham J. (2018). Investigation of the Microstructure Change Due to Phase Transition in Nanosecond Pulsed Laser Processing of Diamond. Carbon.

[16] Fraczyk J., Rosowski A., Kolesinska B., Koperkiewcz A., Sobczyk-Guzenda A., Kaminski Z., Dudek M. (2018). Orthogonal Functionalization of Nanodiamond Particles after Laser Modification and Treatment with Aromatic Amine Derivatives. Nanomaterials.

[17] Jang D.M., Im H.S., Myung Y., Cho Y.J., Kim H.S., Back S.H., Park J., Cha E.H., Lee M. (2013). Hydrogen and Carbon Monoxide Generation from Laser-Induced Graphitized Nanodiamonds in Water. Phys. Chem. Chem. Phys..

[18] Xiao J., Ouyang G., Liu P., Wang C.X., Yang G.W. (2014). Reversible Nanodiamond-Carbon Onion Phase Transformations. Nano Lett..

[19] Cao L., Shiral Fernando K.A., Liang W., Seilkop A., Monica Veca L., Sun Y.-P., Bunker C.E. (2019). Carbon Dots for Energy Conversion Applications. J. Appl. Phys..

[20] Stehlik S., Henych J., Stenclova P., Kral R., Zemenova P., Pangrac J., Vanek O., Kromka A., Rezek B. (2021). Size and Nitrogen Inhomogeneity in Detonation and Laser Synthesized Primary Nanodiamond Particles Revealed via Salt-Assisted Deaggregation. Carbon.

[21] Stehlik S., Varga M., Stenclova P., Ondic L., Ledinsky M., Pangrac J., Vanek O., Lipov J., Kromka A., Rezek B. (2017). Ultrathin Nanocrystalline Diamond Films with Silicon Vacancy Color Centers via Seeding by 2 Nm Detonation Nanodiamonds. ACS Appl. Mater. Interfaces.

[22] Nunn N., Shenderova O. (2016). Toward a Golden Standard in Single Digit Detonation Nanodiamond: Toward a Golden Standard in Single Digit Detonation Nanodiamond. Phys. Status Solidi A.

[23] Stehlik S., Glatzel T., Pichot V., Pawlak R., Meyer E., Spitzer D., Rezek B. (2015). Water Interaction with Hydrogenated and Oxidized Detonation Nanodiamonds—Microscopic and Spectroscopic Analyses. Diam. Relat. Mater..

[24] Petit T., Puskar L., Dolenko T., Choudhury S., Ritter E., Burikov S., Laptinskiy K., Brzustowski Q., Schade U., Yuzawa H. (2017). Unusual Water Hydrogen Bond Network around Hydrogenated Nanodiamonds. J. Phys. Chem. C.

[25] Lawson S.C., Fisher D., Hunt D.C., Newton M.E. (1998). On the Existence of Positively Charged Single-Substitutional Nitrogen in Diamond. J. Phys. Condens. Matter.

[26] Ginés L., Mandal S., Cheng C.-L., Sow M., Williams O.A. (2017). Positive Zeta Potential of Nanodiamonds. Nanoscale.

[27] Boehm H.P. (1994). Some Aspects of the Surface Chemistry of Carbon Blacks and Other Carbons. Carbon.

[28] Stehlik S., Mermoux M., Schummer B., Vanek O., Kolarova K., Stenclova P., Vlk A., Ledinsky M., Pfeifer R., Romanyuk O. (2021). Size Effects on Surface Chemistry and Raman Spectra of Sub-5 Nm Oxidized High-Pressure High-Temperature and Detonation Nanodiamonds. J. Phys. Chem. C.

[29] Chang S.L.Y., Reineck P., Williams D., Bryant G., Opletal G., El-Demrdash S.A., Chiu P.-L., Ōsawa E., Barnard A.S., Dwyer C. (2020). Dynamic Self-Assembly of Detonation Nanodiamond in Water. Nanoscale.

[30] Osswald S., Yushin G., Mochalin V., Kucheyev S.O., Gogotsi Y. (2006). Control of Sp^2^/Sp^3^ Carbon Ratio and Surface Chemistry of Nanodiamond Powders by Selective Oxidation in Air. J. Am. Chem. Soc..

[31] Mermoux M., Chang S., Girard H.A., Arnault J.-C. (2018). Raman Spectroscopy Study of Detonation Nanodiamond. Diam. Relat. Mater..

[32] Mermoux M., Crisci A., Petit T., Girard H.A., Arnault J.-C. (2014). Surface Modifications of Detonation Nanodiamonds Probed by Multiwavelength Raman Spectroscopy. J. Phys. Chem. C.

[33] Stehlik S., Varga M., Ledinsky M., Miliaieva D., Kozak H., Skakalova V., Mangler C., Pennycook T.J., Meyer J.C., Kromka A. (2016). High-Yield Fabrication and Properties of 1.4 Nm Nanodiamonds with Narrow Size Distribution. Sci. Rep..

[34] Osswald S., Mochalin V.N., Havel M., Yushin G., Gogotsi Y. (2009). Phonon Confinement Effects in the Raman Spectrum of Nanodiamond. Phys. Rev. B.

[35] Korepanov V.I., Hamaguchi H., Osawa E., Ermolenkov V., Lednev I.K., Etzold B.J.M., Levinson O., Zousman B., Epperla C.P., Chang H.-C. (2017). Carbon Structure in Nanodiamonds Elucidated from Raman Spectroscopy. Carbon.

[36] Duan X., Tian W., Zhang H., Sun H., Ao Z., Shao Z., Wang S. (2019). Sp^2^/Sp^3^ Framework from Diamond Nanocrystals: A Key Bridge of Carbonaceous Structure to Carbocatalysis. ACS Catal..

[37] Duan X., Ao Z., Li D., Sun H., Zhou L., Suvorova A., Saunders M., Wang G., Wang S. (2016). Surface-Tailored Nanodiamonds as Excellent Metal-Free Catalysts for Organic Oxidation. Carbon.

[38] Reinert L., Zeiger M., Suárez S., Presser V., Mücklich F. (2015). Dispersion Analysis of Carbon Nanotubes, Carbon Onions, and Nanodiamonds for Their Application as Reinforcement Phase in Nickel Metal Matrix Composites. RSC Adv..

