# Supplementary material for: Laser-Induced Modification of Hydrogenated Detonation Nanodiamonds in Ethanol
Source: Nanomaterials (Basel). 2021 Aug 31;11(9):2251. doi: 10.3390/nano11092251 (PMC8472243; doi:10.3390/nano11092251)
Supplement: Supplementary file 1 [file nanomaterials-11-02251-s001.zip › nanomaterials-1351604-SI.pdf]

nanomaterials

Supporting information: Laser-induced Modification of Hydrogenated Detonation Nanodiamonds in Ethanol

Irena Bydzovska [1,2], Ekaterina Shagieva [1], Ivan Gordeev [1], Oleksandr Romanyuk [1], Zuzana Nemeckova [3], Jiri Henych [3,4], Lukas Ondic [1], Alexander Kromka [1] and Stepan Stehlik [1,*]

1 Institute of Physics of the Czech Academy of Sciences, Cukrovarnická 10, 16200 Prague, Czech Republic; bydzovska@fzu.cz (I.B.); shagieva@fzu.cz (E.S.); gordeev@fzu.cz (I.G.); romanyuk@fzu.cz (O.R.); ondic@fzu.cz (L.O.); kromka@fzu.cz (A.K.)
2 Faculty of Nuclear Sciences and Physical Engineering, Czech Technical University, Břehová 7, 11519 Prague, Czech Republic
3 Institute of Inorganic Chemistry of the Czech Academy of Sciences, 25068 Husinec-Řež, Czech Republic; nemeckova@iic.cas.cz (Z.N.); henych@iic.cas.cz (J.H.)
4 Faculty of Environment, J.E. Purkyně University in Ústí nad Labem, Pasteurova 3632/15, 40096 Ústí nad Labem, Czech Republic
* Correspondence: stehlik@fzu.cz

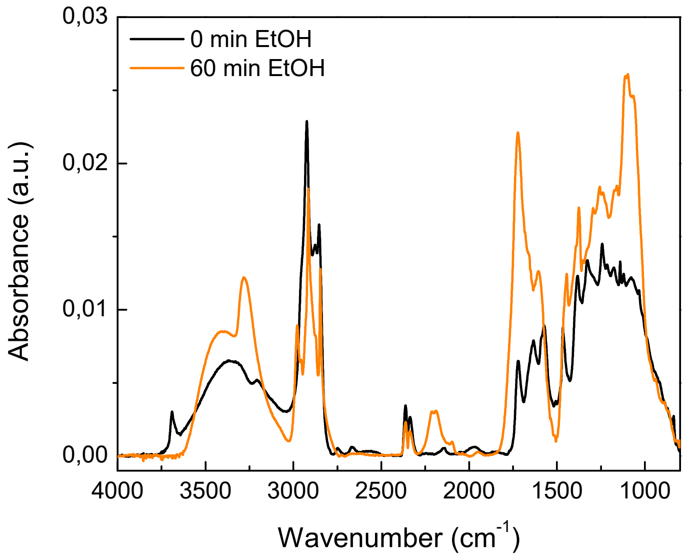

Figure S1. FTIR spectrum of pristine H-DND (0 min; black) dispersed in ethanol and the final yellow solution obtained after 60 min of H-DND irradiation in ethanol (orange).

Figure S1 shows FTIR spectrum of the pristine H-DNDs dispersed and deposited from ethanol (black) and the spectrum of the yellow solution obtained after 60 min of H-DND irradiation. Spectral features of the pristine H-DNDs are very similar to the sample prepared from water, for detailed assignment see the main text. The spectrum of the 60 min sample is different. According to Jang et al. [1] the final yellow solution contain polyyne molecules. The spectral features in the FTIR spectrum indeed confirm the presence of triple C≡C bonds (≈ 2200 cm^{-1}) and ≡C–H alkynes (≈ 3280 cm^{-1}) which might correspond to polyynes. Nevertheless, another chemical bonds (–C–H, C=O,•OH) are also present.

Figure S2 shows HRTEM image of the 40 min sample after its purification by air annealing at 450 °C for 300 min. In correlation with the Raman spectrum (Figure 6e in the

main text), the HRTEM imaging shows typical DND structure, i.e. defective (twins, stacking faults) diamond nanocrystals with dimensions in the 2–8 nm range.

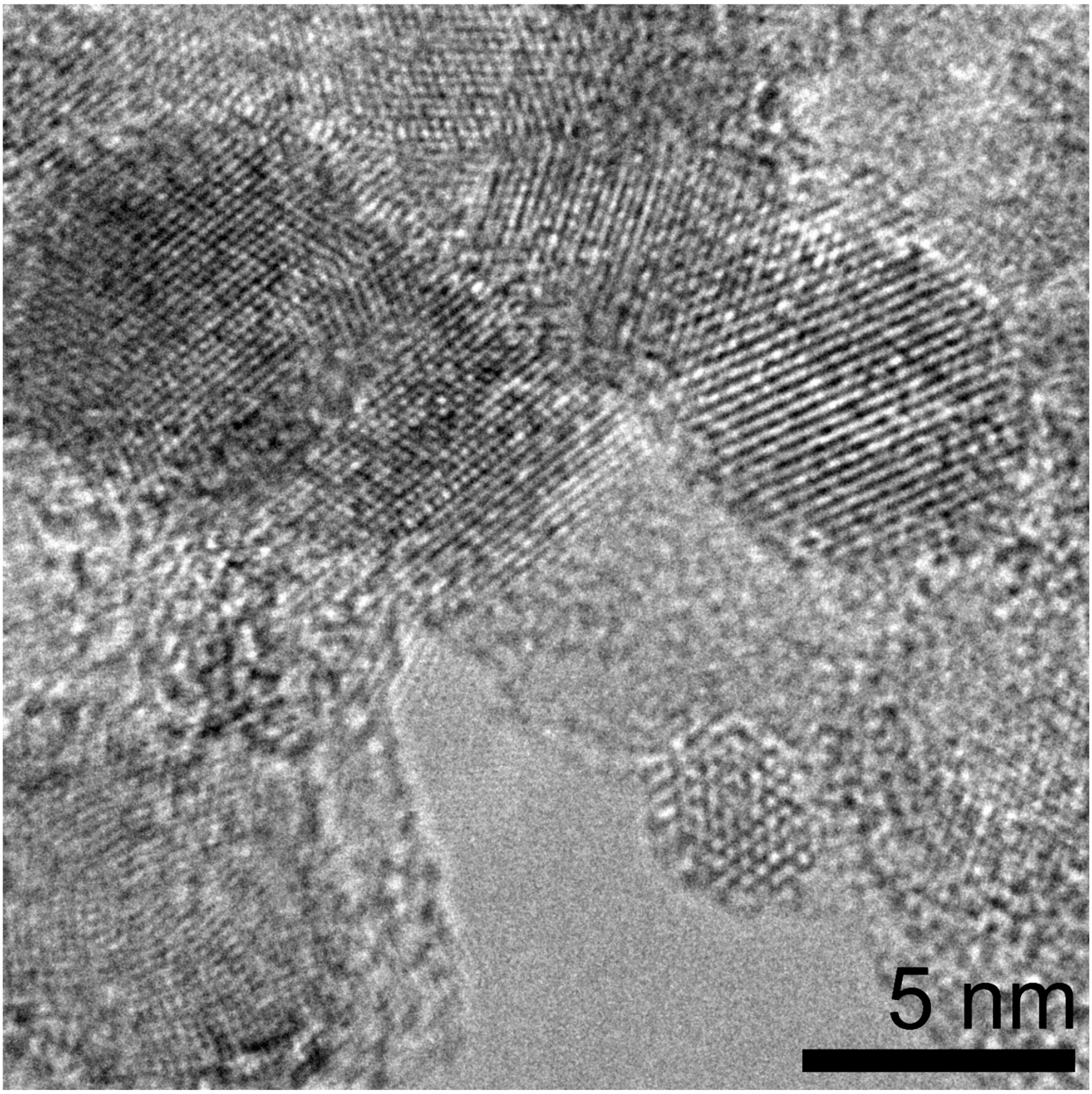

Figure S2. HRTEM image of 40 min sample after its purification by air annealing at 450 °C for 300 min.

We made another set of experiments under the same experimental conditions as in the work of Xiao et al. [2] in order to reproduce the reversible OLC-ND transformation. We dispersed 3 mg of DND powder (New metals and chemicals, Japan) in 10 ml of ethanol by a 1h sonication (Hielscher, UP 200 s) working at 100% amplitude and 50% on/off period. This resulted in good dispersion of the DND powder in ethanol and formation of a greyish colloidal dispersion. Then, the dispersion was irradiated in the same manner as described in the main paper (532 nm, 10 ns pulse width, 10 Hz repetition rate, pulse power of 150 mJ), only the laser beam was focused down to 1 nm to reach the same fluence of 20 J/cm^2 as in the work of Xiao at al [2].

The photograph in the upper part of Figure S3 shows the pristine, non-irradiated sample (0 min) and the samples irradiated for 8, and 24 min. The same evolution from greyish colloid (0 min) across a black-colored (8 min) up to yellow-colored sample (24

min) has been observed as in the main article as well as in the previous works [1,2]. The Raman spectra of the corresponding samples are shown in the lower part of the Figure S3. Again, gradual transformation of DNDs into a graphitic material is observed and no reversible transformation of OLC structures back to ND has been detected. To obtain the same visual appearance of the samples as in the work of Xiao at al [2] the irradiation time had to be shorter, i.e. the transformation rate was obviously higher in our case. This indicates that it is the transformation rate which is quite sensitive to the experimental parameters such as the laser beam parameters as well as concentration and total mass of the irradiated DNDs. On the other hand, our results as well previous results of Jang et al.[1] show that the quality of the obtained material is rather insensitive to the variation of the experimental conditions.

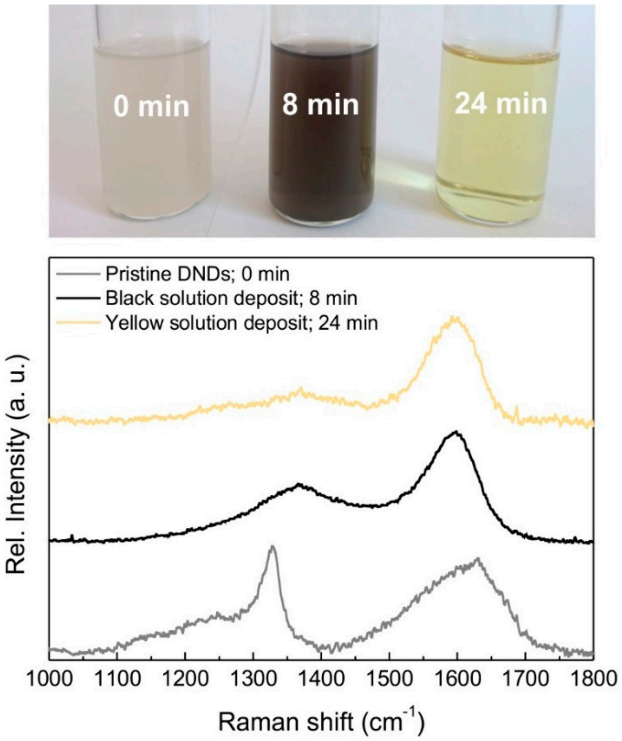

Figure S3. Photographs of the pristine (0 min), and laser-irradiated (8 min, 24 min) samples (upper part). Corresponding Raman spectra of the pristine (grey) and laser-irradiated samples (8 min; black, 24 min; yellow).

References

1. Jang, D.M.; Im, H.S.; Back, S.H.; Park, K.; Lim, Y.R.; Jung, C.S.; Park, J.; Lee, M. Laser-induced graphitization of colloidal nanodiamonds for excellent oxygen reduction reaction. *Phys Chem Chem Phys*. **2014**, *16*, 2411–2416. https://doi.org/10.1039/C3CP54039A.
2. Xiao, J.; Ouyang, G.; Liu, P.; Wang, C.X.; Yang, G.W. Reversible nanodiamond-carbon onion phase transformations. *Nano Lett.* **2014**, *14*, 3645–3652. https://doi.org/10.1021/nl5014234.
